# The Vaccination Process against the COVID-19: Opportunities, Problems and *mHealth* Support

**DOI:** 10.3390/healthcare9091165

**Published:** 2021-09-06

**Authors:** Rossella Simeoni, Giovanni Maccioni, Daniele Giansanti

**Affiliations:** 1 Faculty of Medicine and Surgery, Catholic University, San Martino al Cimino, 010130 Viterbo, Italy; rossella.simeoni.1955@gmail.com; 2 Centre Tisp, Istituto Superiore di Sanità (ISS), Via Regina Elena 299, 00161 Rome, Italy; giovanni.maccioni@iss.it

**Keywords:** COVID-19, SARS-CoV2, *mHealth*, mobile technology, *digital divide*

## Abstract

The vaccination against the COVID-19, finally available, has the potential to represent an important defence against the pandemic. The identification of both obstacles and tools to combat them are, at this moment, of strategic importance. Previous experiences on vaccinations have shown solutions and paths to take, also based on the behavioural sciences. The objective of the *opinion* is to face how mobile technology can help us both to fight these problems and to optimize the vaccination process. The *opinion* has four polarities. The *first polarity* consists in having detected the problems hampering an effective vaccination process. These problems have been grouped into the following four: *Electronic and Informatic divide*, *Escape*, *Exposure risk*, and *Equity*. The *second polarity* consists in having verified how the mobile technology can be useful to face the identified problems. The *third polarity* highlights the usefulness and importance of using electronic surveys. These tools are based on mobile technology. They are useful problem sensors for the stakeholders. The *fourth polarity* faces how mobile technology and *mHealth* can be of aid to optimize the flow of the vaccination process, from the first call up to the certification. This polarity is supported by an example based on the Italian national App IO. The study highlights: (a) on one side, the potential of mobile technology; on the other side, the need for interventions to reduce the *digital divide* with the purpose to increase its use. (b) How the role of mobile technology can be complementary to other intervention methods.

## 1. Introduction

### 1.1. Background

COVID-19 is still prevalent. It is still a terrible threat all over the world. As of 4 August 2021, COVID-19 is the cause of 199,466,211 confirmed cases with 4,244,541 confirmed deaths [1]. Until now, the COVID-19 vaccine is the most promising measure to placate contagion with the hope to reach herd immunity [2].

Unfortunately, public vaccination intention does not appear to be uniform [3]. There are many issues hampering a harmonious and optimized process of vaccination. This was shown through large-scale studies, in which probing tools were also used [4,5,6].

A clear division, for example, exists between medical professionals and laypeople. While the insiders to the healthcare eagerly promote the vaccination campaign, some laypeople show doubt, hesitancy, opposition, and hostility toward COVID-19 vaccines [7]. 

The contrast of these issues is important. It is currently one of the main objectives of politicians and stakeholders of the health domain. It is particularly important to find adequate tools to combat these problems. It is also important to find adequate solutions to optimize the vaccination process itself. 

Scholars have known, even before the pandemic, that adherence to vaccination processes can be improved through: (I) the knowledge of the problems; (II) the use of special tools and precautions to prevent and/or minimize them [8,9].

Jarret et al. [8] highlighted the following useful tools: the use of social mobilization, mass media, communication tool-based training for health-care workers, non-financial incentives, and reminder/recall-based interventions.

Troung et al. recently found in their study [9] that seven major factors promote vaccine hesitancy or acceptance: demographic factors (ethnicity, age, sex, pregnancy, education, and employment), accessibility and cost, personal responsibility and risk perceptions, precautionary measures taken based on the decision to vaccinate, trust in health authorities and vaccines, the safety and efficacy of a new vaccine, and lack of information or vaccine misinformation. They concluded that this approach was useful for strategies to address the present situation with the COVID-19 pandemic. These two studies [8,9] highlight that adherence to vaccination processes depend on many wide-ranging factors also related to the behavioural sciences, such as psychology, sociology, communication sciences.

In particular, communication plays a key role for both the studies [8,9]. Kaufman et al. [10] focused on communication aspects related to vaccines. They reported on the importance of the *face-to-face* interaction in this field. It is an activity playing a basic role. However, now it cannot be played in an adequate way due to the restrictions.

There are many tools dedicated to the communication that can help us to fight these problems. Furthermore, these tools can also improve the vaccination process. Through radio, television, and journalistic communications, in fact, we are all witnessing the dissemination of messages with the aim of raising awareness among citizens. Certainly, the use of mobile technologies, based essentially on the smartphone [11] as a communication tool is an important novelty, compared to previous pandemics (for example the Sars-Cov-1). It is, therefore, of prime importance to investigate its role in this context.

In this study, we dedicated ourselves to an *opinion* on the possible positive role of *mobile technology* (*mTech*) and the *mHealth* both to fight the problems, hampering the vaccination process, and to optimize the vaccination process itself.

*mTech* and *mHealth* have helped [11] us so much during this pandemic. They are still helping us so much in the following three distinct activities: (a) remote monitoring and therapy; (b) continuity of daily life activities through chat, video conferencing, and electronic connection tools; and (c) new epidemiological monitoring opportunities, such as the digital contact tracing. It is therefore important to face how these technologies can help us now, during the vaccination process.

### 1.2. Purpose of the Study

This *opinion piece* has the general objective of highlighting the potential support of *mTech* and *mHealth* in the vaccination process: to combat the various obstacles to an effective and rapid vaccination process; and to optimize the vaccination process itself.

In detail, the work has as its objective to: (a) Directly highlight the main categorized problems related to the vaccination process. (b) Directly highlight what can be the usefulness of *mTech* and *mHealth* both to combat the main problems and to optimize the vaccination process. (c) Indirectly reaffirm the importance for the citizens to connect to the *Health Domain.*

### 1.3. Organization of the Study

The work is organized in four sections (plus the introduction and the conclusions). 

Section 2 reports the main problems hampering the vaccination process.

Section 3, through the evidence from the literature and field evaluations, addresses the objective to highlight how *mHealth* and *mTech* can be of aid to fight these problems. In line with the article type, this section highlights the authors’ strong position regarding the importance of polls in this difficult battle.

Section 4, through an Italian example, highlights how *mHealth* and *mTech* can be useful in optimizing the vaccination process. 

## 2. The *Digital Divide*, the *Escape*, the *Exposure Risk*, the *Equity*, and COVID-19

### 2.1. General Considerations

The effectiveness of a vaccination process depends on the ability to reach all citizens according to principles of fairness, respecting the priorities.

It is therefore of primary importance to meticulously achieve connecting the citizens to the vaccination process. It is also of primary importance to avoid the *escape*, the *hesitancy*, and the *loss* of citizens.

**Figure 1:**
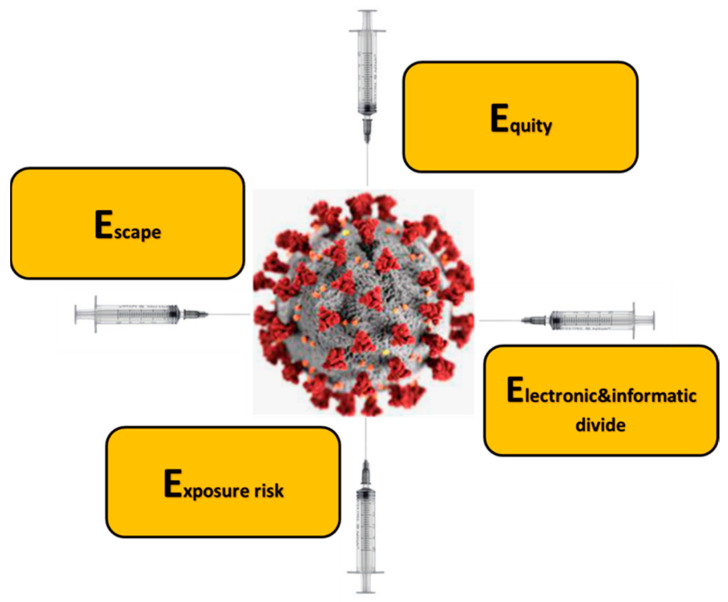
The problems hampering the COVID-19 vaccination process, as identified in our opinion piece.

We must also bear in mind that in the era of digitalization the vaccination processes are particularly based on digital technologies. Therefore, the ability and possibility to connect and/or to join these technologies is strategic. We considered the above, the problems identified by Troung et al. [9], and the role of the priority based on the risk. We identified in this opinion piece a categorization arranged into four issues. They all start with E (Figure 1).

### 2.2. Electronic & Informatic Divide

The *digital divide* in the two components *Electronic and Informatic Divide* regards the gap between those who have effective access to information technologies and those who are partially or totally excluded from it. The *digital divide* has three polarities/levels of intervention. The *first level* of the *digital divide* is represented by the difficulty in the access to the *infrastructures*; this today remains a problem also in the richest and most technologically advanced countries in the world [12]. The *second level* is represented by *the literacy*, characterized by the skills that enable individuals to seek, understand, and use information in ways which promote and maintain health based on digital health [13]. The *third level* is represented by the *potential benefit level* [14]. This regards the extent to which economic, cultural, social, and personal types of engagement with the Internet result in a variety of economic, cultural, social, and personal outcomes. During the COVID-19 pandemic, the problem of the *digital divide* has been exacerbated [15].

The three levels of the *digital divide* are evident also during the pandemic [15,16,17], where digital resources were fundamental [11,15,16,17,18]. The difficulties of the citizens in accessing the digital technologies are multifaceted [15]. They may depend on cultural, ethnic, social, national, and political factors [19,20]; furthermore, they could exacerbate the disparities [20]. The vaccination process is also based (or will be based) on the use of both electronic and computer technologies, with the purpose to achieve effectively the citizens. The *literacy* with the electronic devices, such as tablets, smartphones, and available software—as, for example, with the Italian national App (App IO) (https://io.italia.it/ accessed on 4 September 2021) [21]—could make the difference in the access to vaccines.

### 2.3. Escape

Vaccine *escape* certainly represents an important problem. The lack of confidence in technologies can lead to being forgotten or can lead to not being sufficiently able to join the digitized vaccination process [22]. There is certainly a strong correlation with the problem described above. However, the *escape* from vaccines is also caused by many other factors, such as: (a) the *infodemic* [23], i.e., the pandemic of bad and/or distorted information campaigns; (b) the membership in groups with special beliefs (for example, against vaccines); (c) other diversified multifaceted motifs to be carefully investigated. These problems have a strong impact on the person’s behaviour; furthermore, they must be addressed through suitable tools from the sciences of communication, psychology, and sociology. With the *escape*, we also have the risk of losing those with the greatest risk from exposure, such as frails, who are at risk of becoming seriously ill, as described in the following.

### 2.4. Exposure Risk

The risk of exposure includes two categories: (a) The risk of those who are particularly exposed to viral contamination events, due to work activity, such as healthcare workers or other categories in contact with the public; and (b) the risk of those who, due to their conditions of frailty towards COVID-19, have the potential to contract the disease in a severe form. Prioritization policies must take these aspects into account. It is easy to verify how studies are addressing the issues related to the vaccine assignment priority, based on different hypotheses [24,25,26]. They consider the risk of exposure and other accompanying factors, such as the maintenance of anti-contagion measures, also using simulation models [27].

### 2.5. Equity

*Equity* is a term that, when it is presented into the health domain [27], assumes an important meaning. This meaning is strongly reaffirmed in the pandemic period [28]. *Equity* in the health domain is when everyone can be as healthy as possible. This is achieved by decreasing social disparities and investing more in those who have less, to give everyone the same opportunities. When we talk about the COVID-19, the impact of inequality is immediate because we immediately see that it is synonymous with the continuity of the spread of the SARS-CoV-2 virus, with the difficulty of caring for all citizens, with the difficulty of preventing the disease through vaccination. It is for this reason why many nations are placing the emphasis on this problem through appropriate strategies [29,30].

Therefore, the vaccination process cannot ignore the concept of *equity* as it is interpreted in the health domain. Research in PubMed with the query “(equity [Title]) AND (COVID-19)” showed that the problem of equity is strongly perceived from different points of view, starting from the ethics up to the social vision [31,32,33,34,35,36,37,38,39,40,41,42,43]. 

## 3. The Role of Mobile Technology and *mHealth* to Fight the Problems Hampering the Process

### 3.1. General Considerations

In this section, through the evidence from the literature and field evaluations, we address the objective to highlight how *mHealth* and *mTech* can be of aid to fight the obstacles. In general, *mTech* tries to reach citizens both to bring them closer to the vaccination process and to avoid the *escape, the hesitancy, and the loss*. There can be dedicated apps (which can also use artificial intelligence algorithms) or web sites, with accessibility through *mTech,* designed to fight disinformation (e.g., the infodemic). They may: (I) provide answers to FAQs; (II) inform adaptatively about the priorities in the process (e.g., the frail people); (III) fight the *digital divide*; or (IV) simply try to reach a large population equally. We report below some examples developed around the world in this direction. However, *mTech* also allows us to reach the citizens with tools, such as surveys, helping the stakeholders in the health domain to investigate, in a targeted manner, the position of citizens towards these issues. Our opinion is that their use is important and strategic, since they can allow the creation of large virtual focus groups on important issues. In line with the article type, this paragraph also highlights the authors’ strong position regarding the importance of polls in this period.

### 3.2. Example of mTech Applications

The contribution in the vaccination process of *mHealth* is undisputed by now. Each technologically advanced nation is organizing itself through solutions based on *mTech* and *mHealth* to meticulously achieve the vaccination of citizens and to avoid the *escape,* the *hesitancy,* and the *loss* of people.

Apps can be useful to affect COVID-19 vaccine *hesitancy, escape, and other* related problems. In [44], a national experience performed in Japan is reported. The authors assessed an application (App) based on *mTech*. They successfully assessed a COVID-19 vaccine information *chatbot* inserted in a popular Japanese messenger app (*LINE*). This free app (*Corowa-kun*) aimed to automatically answer FAQs related to the vaccines.

Additionally, artificial intelligence integrated into *mTech* can be useful to fight the vaccine hesitancy, escape, and other problems. The deluge of unverified information, which spreads faster than the epidemic itself, is an unprecedented phenomenon that has put millions of lives in danger. In fact, this has the potential to alter people’s behaviours by making them lose their lucidity.

In ref. [45], the authors describe their created app named *WashKaro*, allowing a multi-pronged intervention for mitigating misinformation through conversational interactions based on artificial intelligence (AI). 

The app *WashKaro* offers correct information in line with the WHO guidelines (including vaccines). It uses AI and delivers information in the right format in local languages. The authors successfully tested it on a wide sample of citizens. 

As we have highlighted, the *digital divide* and the *escape* have connection points.

It is quite evident that the lack of literacy with IT generates an *escape*, but also the inability for those with economic problems to access the IT infrastructures, due to the inability to guarantee *equity*, which also is related to an *escape*.

The Italian government provided, during the pandemic, economic financial aids [46] aimed at equipping the less wealthy people with IT (essentially with the mobile technology, with digitalization kits and smartphones).

This had the clear intention of minimizing the *first level* of the *digital divide*. As it is well known, in rich nations, this has shifted from the difficulty to the physical access to the difficulty in the material access [12]. 

The *escape*, as we have anticipated, also depends a lot on disinformation (e.g., the infodemic). 

The Italian NIH has provided a repository of medical information on its web site with the access also in non-desktop/mobile mode to dispel false news about the vaccine [47]. 

The *escape* also depends on how the institutions reach the citizens through *mTech*. Subtleties in the composition of messages and the language used to reach them are therefore also important to affect their behaviour [48]. In [48], the authors presented two sequential randomized controlled trials (RCTs) that tackle this challenge with behavioural science insights. In the first RCT, text messages designed to make vaccination salient and easy to schedule boost appointment and vaccination rates by 86% and 26%, respectively. Nudges that make patients feel endowed with the vaccine heighten these effects, but addressing vaccine hesitancy via a video-based information intervention does not yield benefits beyond simple text. In the second RCT, they further find that receiving a second reminder boosts appointment and vaccination rates by 52% and 16%, respectively. Their findings suggest that text-based nudges can substantially affect the behaviour and therefore increase and accelerate COVID-19 vaccinations at almost zero marginal cost, highlighting the promising role of behavioural science in addressing a critical component of the COVID-19 pandemic response.

### 3.3. The Usefulness of Dedicated Electronic Surveys Based on mTtech in the COVID-19 Era

The surveys, which today can travel electronically through *mTech,* represent an important tool in the hands of stakeholders to monitor problems. We discussed in [15] that, using accurate diffusion solutions, we can minimize the *digital divide* bias and reach a large population. The surveys have proved to also be highly effective in relation to the pandemic. It clearly emerges that the survey tools proved to be useful for investigating: the impact of bandwidth limitations [49]; the attitude, knowledge, and practice towards COVID-19 [50]; the learning methods [51]; the *equity* and the *digital divide* in the racial and ethnic differences in the comparisons of posts shared on COVID-19 [52]; the *equity* and the *digital divide* in the racial and ethnic differences in the areas of remote assistance during the COVID-19 pandemic [53]; and the impact of the electronic and informatic divide based on age [15].

A recent study in the UK proposed a survey to investigate the *escape* from vaccines [22]. Our opinion is that electronic surveys can be useful to combat the four problems described in Section 2.

The US experience with the electronic surveys [54] conducted through *the U.S. COVID-19 Trends and Impact Survey, 2020–2021* is also very important. This survey is a tool that allows a continuous real-time measurement and monitoring of problems related to the COVID-19. It has operated since 6 April 2020. It is an internet based electronic tool. It operates by inviting a random sample of Facebook active users each day. Over 20 million responses have been received in the first year of operation. The survey has been repeatedly revised to respond to emerging public health priorities. The last revisions are also facing the problems hampering the vaccination process. The study [54] highlighted that large online surveys can provide continuous, real-time indicators of important outcomes for the health domain that are not subject to reporting delays and backlogs.

#### 3.3.1. Experiences and Considerations in the Vaccination Process

As demonstrated in the study in the UK [22] and in the US [54], the surveys represent a powerful tool to investigate the reasons that lead citizens to escape from vaccines. 

As the English and US study show: (I) the COVID-19 pandemic is an experience never tried previously; (II) considering this, it is difficult to find ready-made intervention models to inspire; (III) surveys must also be constructed considering the novelty of the experience we are living in; furthermore, the available mobile technologies allow a widespread dissemination.

In line with the objectives of the opinion piece, we wanted to highlight: (IV) the usefulness of these tools in the widespread data-collection; and (V) the potential usefulness for politicians and decision makers in general. 

Always in line with our objectives, we also wanted to start a scientific discussion in this area. We believe that: (VI) these surveys must be wide-ranging and embedded in the national realities where they are applied, where ethnic and cultural factors can have a significant impact; (VII) they are tools subject to *bias* due to difficulties to access to technology (e.g., *digital divide*). However, it has also been verified in previous studies [15], how some insights in the submission phase allow us to limit the impact of this *bias*. For example, a *peer-to-peer* diffusion with multimedia tools with a clear message to support the less accustomed to IT is useful [15]. 

#### 3.3.2. The Proposal and Test of an Electronic Survey

We designed and validated a wide-ranging electronic survey (eS). We have calibrated it on the Italian national reality. In the introduction, as in [15], we have clearly suggested to support those less familiar with the IT.

As for the questions related to the *escape*, we were inspired by the survey in [22].

Our survey was accompanied with other questions (for example, open questions and/or free questions) to obtain feedback that gives an idea of the critical issues with regards also to the *Equity*, *digital divide* and the *exposure risk*.

The survey was submitted to a first sample to get an initial feedback on its effectiveness and the ability to bring out any critical issues through mobile technology. This survey was tested in the period 1–25 March 2021 on a first sample of citizens, and we are currently evaluating how to transfer it to stakeholders in the health domain, for a structured and broader use. For the test, we focused to a *mission critical* sample. The first test sample selected was represented by the elderly, who represent [15] the persons with the greatest criticality towards the technology: *invited*: *155*; *participated*: *153*; *males/females*: *77/76*; *min age/max age*: *66/77*; *averaged*: *73.3*. 

We then subsequently subjected this survey to an acceptance validation/assessment on two different samples, using some parameters related to the theme of this study (Table 1). Among the *mission critical* persons, in the case of the continuation of a high state of infection with Sars-Cov-2 due to a failure of the vaccination process, we find health workers. We addressed these persons for the validation/acceptance of the survey. Therefore, the two samples were selected considering their sensitivity, experience, and exposition risk in relation to COVID-19. The first group is represented by technical operators of medical radiology: *invited*: *103*; *participated*: *103*; *males/females*: *50/53*; *min age/max age*: *28/57*; *averaged*: *41.3*.

The second group is represented by biomedical laboratory technicians: invited: 89; participated: 89; males/females: 46/43; min age/max age: 28/56; averaged: 42.3. 

Both groups have had and have, albeit operationally different, an important exposure to COVID-19. The former, for example, has exposure during confirmation chest radiology. The second has an exposure from sampling to analysis of the samples.

A comparative opinion of them is, of course, important, even if it is not exhaustive (it would be necessary to hear politicians, decision makers, economists, and other figures), in fact, it gives us an important idea of who struggles every day with this problem, in line with a piece of opinion. No technical problems were reported.

In [55], the electronic survey is available. As for the questions (*Q21, Q22, Q24*) on the intention or non-intention to receive the vaccine, we were inspired by the survey in [22]. Furthermore, different types of questions were used (*choice, multiple choice*, *Likert*, *open*, *graded evaluation*) to face the themes related the *escape*, the *exposure risk*, the *equity*, and the *digital divide* [55]. In relation to all those who participated in the survey (153 out of 155) the number of 129 (84.31%) showed their intention to be vaccinated, while the number of 24 (15.69%) no, a percentage that if confirmed in wide-ranging investigations would be decidedly worrying. The two main reasons for no are in order: the concern for future effects (24 votes), the concern for side effects (4 votes). The two groups involved in the eS validation process were free to try the survey after receiving it. Table 1 highlights the highly positive opinion on the survey on the first group of assessors. The Shapiro–Wilk test, which is preferable for small samples, successfully tested the normality on the two groups.

The answers of this second sample of assessors did not differ significantly from those of the first in average (T-student, *p* > 0.5 in all the comparisons among the averaged values).

## 4. The *mTech* and the Usefulness to Optimize the Process: The Italian Example

### 4.1. General Considerations

The vaccination process is benefiting from *Digital Health* for the optimization of the path, from the first call, based on predefined priorities, to the issuance of the certificate. The two key components of *Digital Health* are the electronic health (*eHealth*) and *mHealth* (which uses *mTech*). 

The *eHealth* is fundamental for many activities, such as, for example, in the connection between the Hospital Information System and national networks (with medical records and data). 

The *mHealth* is essential to connect the citizen to the process through *mTech.* In many countries with a fair degree of technological evolution, there are (national) apps connecting citizens to the public administration. 

These Apps allow access through the so-called digital identity. They have been equipped, in some cases, with useful functions for Digital Health applications. These functions also allow the management of the vaccination process in an optimized way.

### 4.2. An Example of the Italian Approach

In Italy, the booking processes are also based on the national *App IO* [21]. The app of the public services uses an access based on the *digital identity*. Aware of the problem of the *digital divide* (Section 2.2) from a literacy point of view [13], the Italian state, during last autumn, activated familiarization initiatives towards this app by inserting a reimbursement program (Cashback) [56].

The program managed by the app allows a reimbursement for most of payments made by electronic money. This has made it possible to decrease the *digital divide* from a literacy point of view and to arrive more ready for the vaccination process also managed by this app.

Basically, in Italy, *Digital Health* also uses this App in the vaccination process.

**Figure 2:**
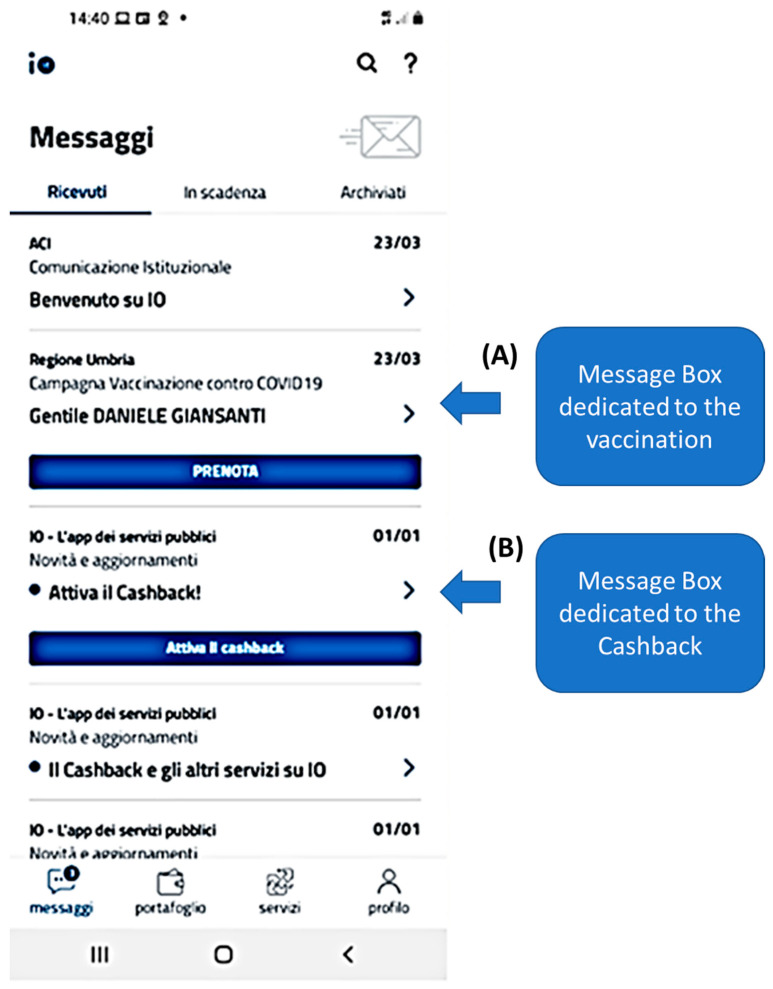
The message box dedicated: (**A**) to the vaccination program (**B**) the Cashback.

**Figure 3:**
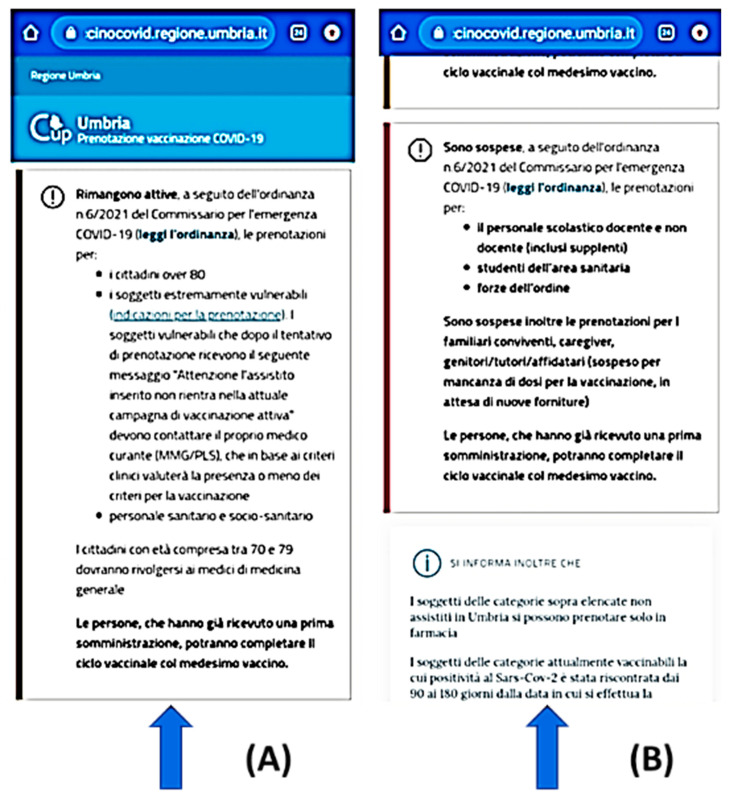
The message box highlights an example of the priority changes during the last spring following the legislative changes (**A**) (citizens over 80; frail people; personnel in the health domain). (**B**) Information on the changed priority during the submission (teachers, police and army personnel, care-givers and relatives of frails are excluded).

**Figure 4:**
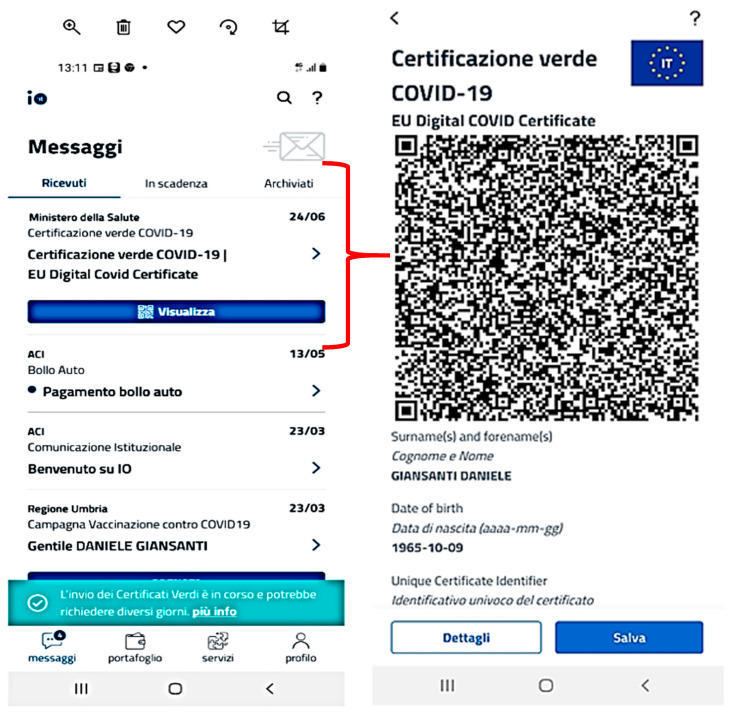
The message box related to the EU COVID-19 vaccine certificate (**left**) and the final EU certificate, with the English translation (**right**) after the download.

It is also through this App that priority processes related to vaccination can be managed. There is the possibility to access various sections of a message box (Figure 2). Figure 2, Figure 3 and Figure 4 reports for the message box of an author (Giansanti D).

Figure 2, A reports the message box of the vaccination process. Figure 2, B shows the message box of the reimbursement program.

Figure 3 reports the message box highlighting an example of the priority changes during the last spring, following the legislative changes. The active priorities are shown in the section A. They consider the exposure risk, dynamically updated based on national models and regulations. The screenshot shows a change in priority as reported in section B. Figure 4 shows the end of the process manged by the app with the production of the EU certificate (*Green Pass*), subtitled with an English translation available in the message box.

## 5. Conclusions

### 5.1. Highlights

The COVID-19 pandemic shows numerous peculiarities when it is compared with previous pandemics. We can highlight, among these ones, those relating to the availability of the *mTech*, based on smartphones. While it was once necessary to develop dedicated wearable technological solutions [57] for medical applications, today, the smartphone allows the citizen to interact with the health domain through *mTech* and *mHealth* applications [58]. The important role of this technology has been seen and is continuing to be seen in telemedical applications, such as in the activities related to the continuity of care in the various forms of teleconsultation, television, and tele-diagnosis [59]. We have also seen, during the pandemic, the leading role of these technologies in the most common applications of daily life, from teaching to e-banking [11], or in the innovative digital contact tracing applications [60].

The *opinion* has addressed the role of *mTech* in this period, in which mass vaccination is being carried out, and in which incredible efforts are being made both to meticulously reach the citizen and not to lose him.

The experience related to vaccination suggests problems to be faced and solutions. The communication is certainly playing an important role in this moment [8,9]. It has a key role, as it can strongly affect the behaviour of the citizen. However, in this moment, the communication has lost the important component of *face-to-face* interaction [10] due to social distancing. Experts and scholars are undoubtedly facing optimal strategies to positively influence the behaviour, through solutions based on traditional means. We have seen the important role of these tools, for example, in convincing elderly people to get vaccinated [61]. It was also seen how the role of the incentives, offered through these messages, can have a positive impact on the intention to vaccinate, as illustrated for example in [62]. It was highlighted in this study as a message that offers cash money, compared to lottery, is more captivating.

In our study, it has been highlighted how mobile technology can be particularly helpful in this moment on this issue. It was highlighted how specific Apps can play an important role as a communication tool capable of allowing the dissemination of correct information, fighting the *infodemic* [44,45,47], and recalling citizens with calibrated messages [48].

The importance of electronic surveys has also been elucidated in [54], traveling through *mTech,* as sensors for politicians and stakeholders in the health domain. This is the case of the US experience with the electronic surveys [54], through the *U.S. COVID-19 Trends and Impact Survey, 2020–2021*, which had 2,000,000 hits. This national survey has undergone continuous updates, and the latest revisions:

1. *New questions: vaccine intent. Vaccine status item enabled on 6 January 2021, 19 December 2020;*
2. *Textual revisions to vaccine intent items 12 January 2021;*
3. *New questions: Reasons for vaccine hesitancy, vaccine dosing Minor textual revisions 8 February 2021;*
4. *New questions: Appointments for COVID vaccines, information about getting vac-cinated Textual revisions 2 March 2021;*

focused precisely on this theme.

The large-scale usefulness of electronic surveys was also reaffirmed through our brief analysis reported in this study. *mTech* is also today essential in optimizing the vaccination process, following the citizen in all the phases ranging from the first call up to the issuance of the certificate as illustrated through an example based on the Italian IO App [21].

### 5.2. Final Reflections

Both the importance and the potential of the role of *mTech* clearly emerges in the study. However, the importance of not being *cut off* from access to these technologies also clearly emerges indirectly. These difficulties fall within the problem of the *digital divide*. They range from the difficulties in accessing infrastructures up to the degree of literacy [12,13,14].

While in poor countries there is still a physical difficulty in accessing the infrastructure, in rich countries this difficulty is becoming material [12].

Initiatives that improve access to infrastructure are welcome, for example through the provision of economic bonuses for the purchase of IT [46]. Initiatives that improve *literacy* are also welcome, as the initiative that we have reported and described in the study [56].

Through our study, the importance of electronic survey tools also emerges, as in the case of US surveys [54]. It is true that these surveys, as highlighted in [15], using appropriate precautions can allow us to minimize the *bias* due to the *digital divide* (for example, by inviting those most familiar with technology to support the less familiar). It is also true that these biases in general cannot be eliminated. We must therefore not forget other solutions. We must not forget other methods, such as the qualitative methods, which, as it is well known, provide a methodology for the description of phenomena, such as understanding and behaviour [63,64,65]. They are based on inductive reasoning, according to which hypotheses derive from observations. We must not forget other emerging techniques with qualitative output based on the so-called sentiment analysis. The sentiment analysis is a methodology evaluating the movements of opinion on social media, also analysing the activity on Facebook, Twitter, Instagram, Youtube, Twitch, news and blog platforms [66]. This methodology takes into consideration only citizens who interact with social media (not conditioned by the *digital divide*), however, in this way, large movements of opinion are kept under control.

### 5.3. Final Thought

The final thought for the stakeholders in the health domain is that *mTech* can play an important role in this phase. Nevertheless, it remains an important slice of the population with difficulty in accessing the IT, both in rich and in disadvantaged countries. It is therefore necessary to invest in initiatives that minimize the *digital divide*. It is also necessary not to forget that *mTech* will never be able to supplant other methodologies, such as the irreplaceable one of *face-to-face* communication. It will have a useful, but certainly complementary, role.

## Figures and Tables

**Table 1:** Opinions of the 103 insiders on the survey (1 = minimum grade; 6 = maximum grade).

Opinion on:	Averaged Grade
*The survey is capable to face aspects of equity, exposure risk, electronic and informatic divide, escape.*	5.1
*The survey is user-friendly.*	4.9
*The survey is clear.*	4.8
*The survey is fast in the operations.*	4.9
*The survey runs well*	4.9
*The survey is useful for the government*	5.3
